# SOS3 from *Avicennia marina* Enhances Salt Stress Tolerance of *Arabidopsis thaliana*

**DOI:** 10.3390/cells14120935

**Published:** 2025-06-19

**Authors:** Mariam Alzaabi, John Orpilla, Khaled Michel Hazzouri, Ling Li, Khaled Amiri

**Affiliations:** 1 Khalifa Center for Genetic Engineering and Biotechnology, United Arab Emirates University, Al-Ain P.O. Box 15551, United Arab Emirates; zmaryam@uaeu.ac.ae (M.A.); johnoliverorpilla@gmail.com (J.O.); khaled_hazzouri@uaeu.ac.ae (K.M.H.); 2 Department of Biology, College of Science, United Arab Emirates University, Al-Ain P.O. Box 15551, United Arab Emirates

**Keywords:** SOS pathway, SOS3, CBL4, *Avicennia marina*, *Arabidopsis thaliana*, salt stress

## Abstract

Abiotic stress poses a serious challenge in agriculture. Salinity inhibits crop growth and yields by disrupting ionic homeostasis and osmotic balance. One critical mechanism of salt tolerance is the activation of the Salt Overly Sensitive (SOS) signaling pathway. Investigating this pathway in halophytic plants offers valuable insights into the molecular mechanisms underlying salt stress tolerance. This study explores the structure and function of *SOS3*/*CBL4* from the gray mangrove, *Avicennia marina* (*AmSOS3*). Sequence analysis revealed that AmSOS3 shares significant similarities with orthologs of SOS3/CBL4, including *Arabidopsis thaliana* (AtSOS3). All essential functional domains of SOS3, including the four EF-hands, as well as the N-myristoylation and S-acylation motif, were conserved in AmSOS3. Structural modeling, using Modeller, predicted that AmSOS3 forms a homodimer stabilized by a hydrogen bond at the serine 140 position. Functional characterization further demonstrated that *AmSOS3* complements the *sos3-1* mutation in *A. thaliana*, thus confirming that *AmSOS3* is an ortholog of *AtSOS3*. Overexpression of *AmSOS3* in wild-type *A. thaliana* enhanced tolerance under salinity stress. The transgenic lines displayed reduced reactive oxygen species (ROS) accumulation and increased ROS-scavenging enzyme activity. These findings indicate that *AmSOS3* plays a critical role in improving salt stress tolerance and maintaining cellular homeostasis.

## 1. Introduction

Abiotic stress plays a major role in reducing agricultural productivity. A report by the UN Food and Agriculture Organization (FAO) estimated soil affected by salinity to be around 1.4 billion hectares globally, representing around 10.7% of land area [1]. It is projected that by 2050, approximately 50% of arable land will be affected by salinity [2,3]. Salt stress disrupts plant ion homeostasis and osmotic balance, causing diminished growth and reduced crop production [4,5]. Halophytic plants, however, have adapted to grow in environments with increased salt concentrations. These plants have evolved complex physiological and biochemical mechanisms that enable survival in saline environments [6].

One of the key molecular players in salt stress adaptation is the Salt Overly Sensitive (SOS) signaling pathway [7]. The SOS pathway is involved in Na^+^ extrusion during salt stress and is crucial in maintaining cellular ion homeostasis [8]. The core SOS pathway, composed of *SOS1*, *SOS2*, and *SOS3*, was first identified in *Arabidopsis thaliana*. The *SOS1* gene encodes a plasma-membrane-localized Na^+^/H^+^ antiporter [9,10]. *SOS2* encodes a serine/threonine protein kinase [11], and *SOS3* encodes a calcium-binding protein [12]. SOS3, also known as calcineurin B-like protein 4 (CBL4), belongs to the plant-specific CBL family of calcium sensors. Of the ten CBLs in the *Arabidopsis thaliana* genome, SOS3/CBL4 is predominantly expressed in roots, while SOS3-like calcium binding-protein 8 (ScaBP8)/CBL10 is mainly expressed in shoots [13]. SOS3 functions upstream of this pathway by detecting calcium oscillations in response to salt stress. Upon binding to Ca^2+^, SOS3 interacts and activates the serine/threonine protein kinase SOS2 [14,15]. This SOS3–SOS2 interaction facilitates the recruitment of SOS2 to the plasma membrane, where it phosphorylates and activates the membrane-associated Na⁺/H⁺ antiporter SOS1 [16].

Loss-of-function mutations in any of these genes result in salt hypersensitivity in *Arabidopsis thaliana*, whereas overexpression of the SOS genes confers salt tolerance, demonstrating that each gene is essential for salt homeostasis [9,14,17]. Interestingly, the overexpression of *AtSOS3* alone was shown to be as effective as overexpressing all three *SOS* genes combined in *Arabidopsis thaliana* [18]. Recently, a salinity-dependent but SOS2-independent interaction between SOS3 and SOS1 and its recruitment to the plasma membrane has been reported [19]. Furthermore, under salt stress, SOS3 interacts with and directs the degradation of HKT1;1 to prevent Na⁺-unloading from the xylem, thus demonstrating that SOS3 acts as a molecular switch for both SOS1 and HKT1;1 to maintain the Na⁺ balance under salt stress [19]. Overexpression of *SOS3* genes from several halophytic plants, such as *Pongamia pinnata* and *Tamarix hispida*, in *Arabidopsis thaliana* has been shown to enhance salt stress tolerance [20,21]. Moreover, SOS3 was shown to influence actin cytoskeleton reorganization and lateral root development in response to salt stress, highlighting its multifaceted role in plant stress responses [22].

SOS3 is a calcium-binding protein belonging to the EF-hand protein family (CaBPs). The EF-hand motif is a highly conserved calcium-binding domain [23]. This domain consists of a helix-loop-helix structure, where the loop region typically consists of 12 amino acid residues responsible for calcium ion coordination [16]. The conserved sequence for N-myristoylation is MGXXXS/T(K), where the glycine residue at position 2 is essential for SOS3 myristoylation [17]. Targeted substitution of glycine to alanine in AtSOS3 prevented its myristoylation and resulted in a salt-hypertensive phenotype similar to that in the *sos3-1* mutant [17]. In addition, S-acylation of AtSOS3 at the cysteine residue at position three redirects the protein toward the nucleus. Mutation of Cys3 to Ala prevented nuclear localization of SOS3 [24].

The crystal structure of AtSOS3 illustrated that SOS3 forms a homodimer via a short linker [16]. A sedimentation equilibrium study in a Ca^2+^-containing solution showed that SOS3 exists in an equilibrium of monomer/dimer forms. The dimeric form of SOS3 increased with rising Ca^2+^ concentration, establishing that calcium binding promotes dimerization [16].The crystallographic dimer of AtSOS3 displayed a V-shaped conformation, where two monomers were connected by a hydrogen bond between the serine 144 residues (144S) [16].This dimerization is essential for SOS2 activation through conformational change [16].

Halophytic plants have evolved to survive in high-salinity and harsh environments [25]. They provide important genetic resources for understanding plant salt resistance and improving salt tolerance in some crops. Mangroves are halophytic plants that inhabit tropical and subtropical coastal areas [25]. The gray mangrove (*Avicennia marina*) is the dominant species along the coastal green forest of the United Arab Emirates (UAE) [26]. Mangroves typically grow in areas where salt concentrations are below 45 to 50 ppm. However, along the coast of the UAE, gray mangroves thrive at salt concentrations that can reach from 65 to 70 ppm [26]. This makes the gray mangrove an excellent candidate for investigating the salt-tolerance adaptation via genomic and molecular studies. In the present study, we characterized *SOS3* from *Avicennia marina* (*AmSOS3*). Sequence and structure analyses of AmSOS3 based on AtSOS3 revealed significant conservation between them. The important functional domains of SOS3, including four EF-hands and the N-myristoylation domain, are conserved in AmSOS3. The structure prediction of AmSOS3 protein using AtSOS3 as a template showed high similarities between AmSOS3 and AtSOS3, suggesting a comparable function. Overexpression of AmSOS3 in *Arabidopsis thaliana* conferred enhanced tolerance to salt stress. Furthermore, AmSOS3 successfully complemented the *sos3-1* mutant *Arabidopsis thaliana*, confirming its functional conservation to AtSOS3 and highlighting its role in stress resilience. This study presents insights into the structure and function of SOS3 from the halophyte *Avicennia marina* and underscores its potential for developing stress-resilient crops.

## 2. Materials and Method

### 2.1. Amplification of AmSOS3 cDNA and Plasmid Construction

Total RNA was extracted from *Avicennia marina* root tissues using a modified CTAB method [27]. The homogenized root samples were added to CTAB (Cetyl trimethylammonium bromide) buffer (2% *w*/*v*), 2% (*w*/*v*) PVP (Polyvinylpyrrolidone K30), 100 mM Tris-HCl, pH 8.0, 25 mM EDTA (Ethylenediaminetetraacetic acid), 2.0 M NaCl, 500 mg/L Spermidine, and 2% (*v*/*v*) β-Mercaptoethanol. Samples were incubated at 65 °C for 15 min with occasional mixing. Following centrifugation, an equal volume of Chloroform: Isoamyl alcohol (24:1) was added to the supernatant and mixed thoroughly. The RNA was precipitated using 10 M LiCl, washed with 70% ethanol, air-dried, and dissolved in nuclease-free water. The quality and integrity of the RNA were assessed using a NanoDrop spectrophotometer (ThermoFisher, MA, USA) and agarose gel electrophoresis. cDNA synthesis was performed using the Superscript III Reverse Transcriptase Kit (ThermoFisher, USA) according to the manufacturer’s protocol, with 1 µg of total RNA as the template. A pair of degenerate primers was designed based on conserved regions of *SOS3* homologs from related species to amplify a partial fragment of the *AmSOS3* cDNA. The amplified product was sequenced and used to design specific primers for 5′ and 3′ RACE experiments (Rapid Amplification of cDNA Ends) (Takara, Kyoto, Japan). The full-length AmSOS3 cDNA was amplified using specific primers. All primers used are in Table 1. The full-length cDNA was digested with NdeI and SalI and ligated into the plant expression vector pRI201-AN (Takara, Kyoto, Japan), generating the recombinant plasmid pRI 201-AN:35S::AmSOS3. The sequence of the *AmSOS3* cDNA insert was confirmed by Sanger sequencing (Macrogen, Seoul, Korea). Then, it was analyzed and compared with the sequence of *Arabidopsis thaliana SOS3* using the BLAST (Basic Local Alignment Search Tool, BLASTP 2.8.0) online tool (https://blast.ncbi.nlm.nih.gov/Blast.cgi, accessed on 2 February 2017). The Clustal Omega (CLUSTAL O(1.2.4) multiple sequence alignment) online tool was also used to align the AmSOS3 sequence with the homologous sequences from other species to assess sequence conservation (http://www.clustal.org, accessed on 25 September 2024).

### 2.2. Analysis of AmSOS3 cDNA Sequence and Protein Prediction

Jalview software (version 2.11.4.1) was used to align the protein sequence of AmSOS3 with all known Calcineurin B-like (CBL) proteins (CBL1–CBL10) from *Arabidopsis thaliana*. The *Arabidopsis* CBL protein sequences were retrieved from the UniProt database with the following accession codes: O81445, Q8LAS7, Q8LEM7, O81223, Q7FZF1, Q9C5P6, Q9SUA6, Q9FUQ7, Q9LTB8, and Q7FRS8. ClustalW, integrated within Jalview, was used for the sequence alignment. Additionally, a phylogenetic tree was generated using the average distance method and the BLOSUM62 substitution matrix within Jalview. Protein structure prediction of AmSOS3 was performed using MODELLER, with the Arabidopsis SOS3 model as a template [28]. The predicted protein structure was then visualized, and images were generated using ChimeraX software (version 1.8) [29].

### 2.3. Salt Stress of Transgenic Arabidopsis thaliana

Wild-type (WT) *Arabidopsis thaliana* (Columbia ecotype, *Col-0*) and *sos3-1* mutant plants were transformed with the pRI 201-AN:35S::AmSOS3 construct using the floral dip method [30]. The T1 transformants were selected on half-strength Murashige and Skoog (½MS) medium containing 1% sucrose and kanamycin and subsequently confirmed by PCR analysis. To assess salt stress tolerance, 7-day-old T4 seedlings were grown on ½MS plates with 1% sucrose supplemented with varying concentrations of NaCl: for overexpression in wild-type (0, 125, or 140 mM NaCl), for overexpression in mutant plants (0, 50, or 100 mM NaCl). Additionally, salt stress assays were conducted in the soil. Two-week-old plants were watered with 100 mL of 150 mM NaCl solution every other day for three weeks, and the survival rates were evaluated. Plants exhibiting sustained wilting, chlorosis, or necrosis after the recovery period were classified as non-survivors. The salt experiment was conducted with 12 biological replicates.

### 2.4. RT-qPCR of Arabidopsis thaliana and Avicennia marina

Wild-type and T4 transgenic *Arabidopsis thaliana* seeds were grown in soil for 10 days before being transferred to a hydroponic system (1/10-strength MS) for acclimatization over 5 days. Plants were then subjected to salt stress in 1/10-strength MS medium supplemented with 150 mM NaCl for 24 h. Leaves and root tissues were collected after 24 h, and total RNA was extracted using the Maxwell^®^ RSC instruments and Maxwell^®^ 48 kits (Promega, Wisconsin, US). cDNA synthesis was performed using the QuantiTect Reverse Transcription Kit (ThermoFisher, Massachusetts, USA). Quantitative PCR (qPCR) analysis was performed using Fast SYBR™ Green Master Mix (Applied Biosystems, Massachusetts, USA) following the manufacturer’s protocol. Gene-specific primers (Table 1) were used to assess the expression levels of *AmSOS3*, with Actin serving as the internal housekeeping gene for normalization. Relative gene expression was calculated using the ΔΔCt method [31].

For *Avicennia marina*, three months old plants were grown in hydroponic system of 1/10 MS basal medium, pH 6, containing 150 mM NaCl for two weeks to acclimatize the seedlings. For salt stress, the concentration of NaCl in the medium was increased to 500 mM NaCl. Leaf, main root, and lateral root samples were collected after 4 h and 24 h, and homogenized in liquid nitrogen. CTAB method was used to extract RNA. The following steps are the same as *Arabidopsis thaliana*. For *Avicennia marina* qPCR, elongation factor (EF) gene was used as the internal housekeeping gene.

### 2.5. Biochemical Staining

Reactive oxygen species (ROS) accumulation was assessed by histochemical staining using 3,3′-diaminobenzidine (DAB) and nitro-blue tetrazolium (NBT) following the protocol described in [32]. For DAB staining, leaves were immersed in a freshly prepared 1 mg/mL DAB solution (pH 3.8) and incubated overnight at room temperature in the dark. The next day, leaves were cleared by boiling in 95% ethanol for 10–20 min and stored in fresh ethanol until imaging. DAB staining detects hydrogen peroxide (H_2_O_2_) accumulation, which appears as brown precipitates. For NBT staining, leaves were incubated in a 0.1% (*w*/*v*) NBT solution prepared in 50 mM sodium phosphate buffer (pH 7.5) overnight at room temperature in the dark. After staining, the leaves were decolorized by boiling in 95% ethanol for 10–20 min and stored in fresh ethanol. NBT staining detects superoxide radicals (O_2_^−^), which appear as blue precipitates.

### 2.6. Enzymatic Assay

Peroxidase concentration was measured using the protocol by Arnnok et al. [33]. Catalase (CAT) levels were measured using a modified protocol of Cakmak and Marschner [34]. Superoxide dismutase (SOD) levels were measured using Monnet et al.’s protocol [35].

### 2.7. Statistical Analyses

Statistical analyses were performed using Microsoft Excel. Data were compared using one-way ANOVA, and differences were considered statistically significant at *p* < 0.05. An asterisk (∗) indicates a significant difference (*p* < 0.05).

## 3. Results

### 3.1. Sequence Analysis and Phylogenetic Tree Comparison of AmSOS3

**Figure 1:**
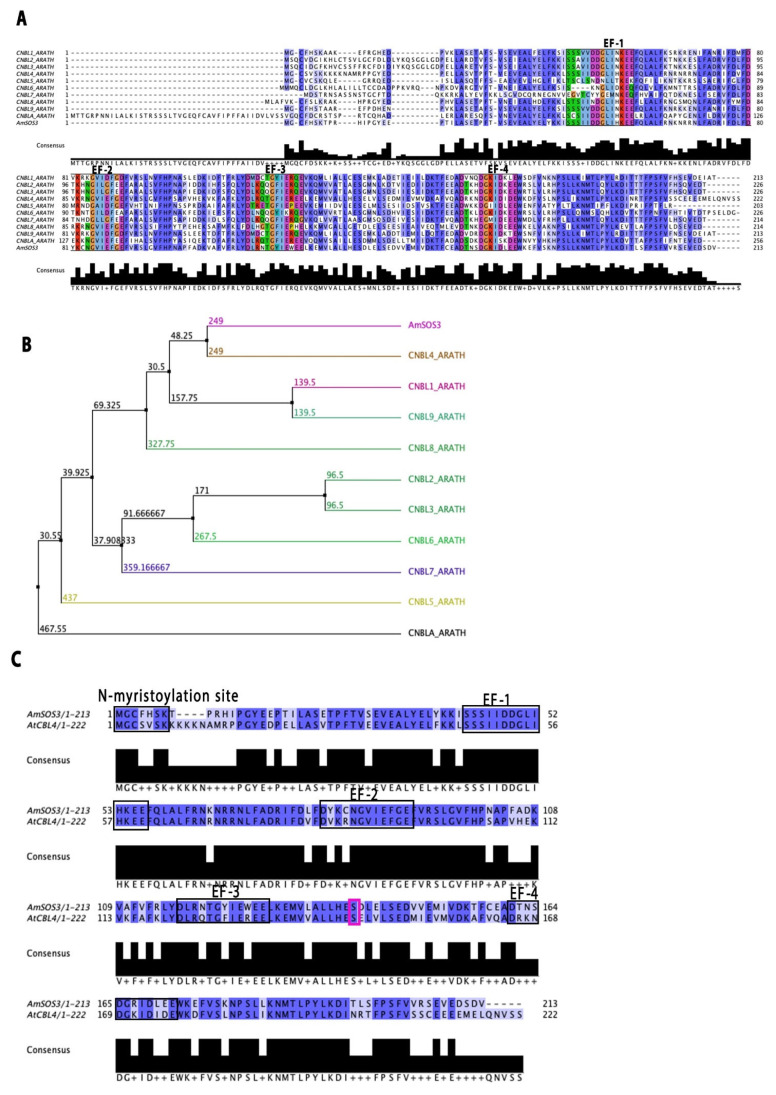
Sequence and phylogenetic analysis of AmSOS3. (**A**) Protein sequence alignment of AmSOS3 against all known Calcineurin B-like (CBL) proteins (CBL1–CBL10) from *Arabidopsis thaliana*. (**B**) Phylogenetic tree constructed using the average distance method and the BLOSUM62 (Jalview). (**C**) Sequence alignment of AmSOS3 and AtSOS3(CBL4), indicating the N-myristoylation domain, the four EF hands, and Ser 140 for dimerization in the pink box.

A partial *AmSOS3* cDNA was amplified from the *Avicennia marina* root using degenerate primers, followed by Rapid Amplification of cDNA Ends (RACE), resulting in a 649-nucleotide cDNA fragment. The open reading frame (ORF) is 642 nucleotides and encodes a protein of 213 amino acids. BLAST analysis of the AmSOS3 sequence revealed high sequence similarity to SOS3/CBL4 from various species, including *Olea europaea* var. *sylvestris* (XP_022887026.1), *Erythranthe guttata* (XP_012852071.1), *Andrographis paniculata* (XP_051143851.1), and *Arabidopsis thaliana* (AT5G24270), with sequence identities of 87%, 83%, 86%, and 71.43%, respectively. The sequence alignment of AmSOS3 with the Calcineurin B-like proteins, CBL1–CBL10, from *Arabidopsis thaliana* revealed significant conservation, particularly in the EF-hand domains (Figure 1A). Phylogenetic analysis, constructed using the average distance method and the BLOSUM62 substitution matrix in Jalview, demonstrated that AmSOS3 is most closely related to AtCBL4/SOS3, as indicated by their close clustering in the tree (Figure 1B). The identity matrix score between AmSOS3 and AtCBL4 was calculated to be 71.43%, reflecting a high degree of sequence similarity.

Interestingly, the EF1-hand domain of AmSOS3 showed 100% similarity with that of AtSOS3/CBL4. The subsequent two EF-hand motifs (EF2 and EF3) exhibited 81.82% and 75% similarity, respectively, while the EF4-hand displayed the least similarity at 50%. Additionally, a conserved N-myristoylation consensus sequence (MGXXXS/T(K) was identified as (MGCFHSK) in AmSOS3 (Figure 1C). In this domain, both the glycine at position 2 (Gly-2), necessary for N-myristoylation, and the cysteine at position 3 (Cys-3) required for *S*-acylation are conserved. Furthermore, the serine residue implicated in dimerization was identified at position 140 (Ser-140), between the EF3 and EF4 domains, highlighted in the pink box (Figure 1C). These findings confirm that AmSOS3 retains the essential structural features of *AtSOS3*. Altogether, the sequence and phylogenetic analyses support the fact that AmSOS3 is an ortholog of SOS3, likely to perform similar molecular functions in salt stress response.

### 3.2. Structural Modeling of AmSOS3

**Figure 2:**
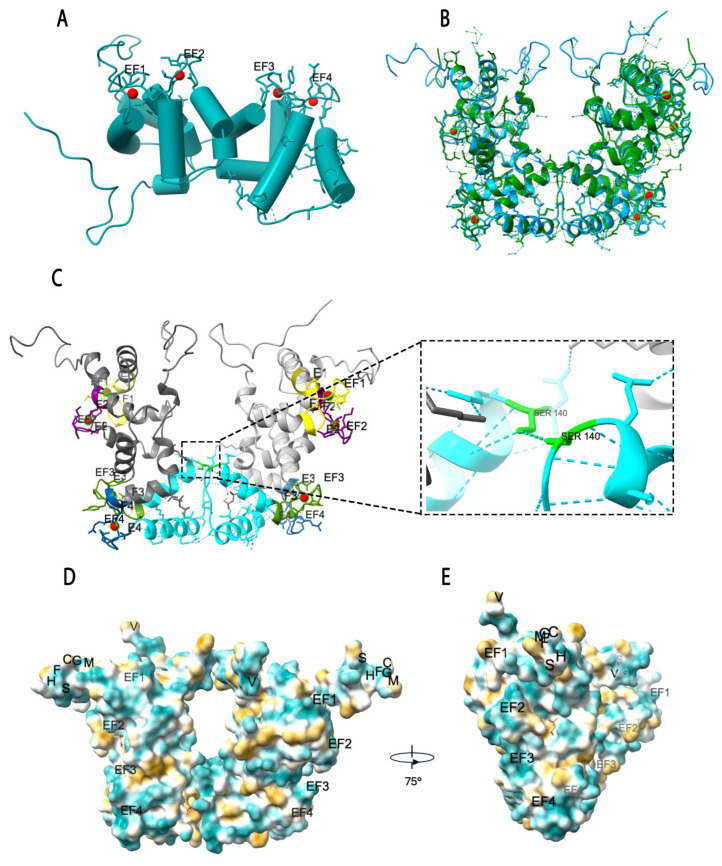
Predicted structure of AmSOS3. (**A**) Predicted monomeric structure of AmSOS3, highlighting four EF-hand motifs essential for calcium binding, calcium ions are in red. (**B**) Superimposition of the dimeric structures of AmSOS3 (cyan) and AtSOS3 (green), (**C**) A view of AmSOS3 protein indicating each EF-hand, EF1 (yellow), EF2 (purple), EF3 (green), EF4 (blue), and a close-up view of the dimerization interface, where a hydrogen bond between serine 140 residues stabilizes the dimer. (**D**) Hydrophobicity view of AmSOS3, illustrating key structural elements, including the N-myristoylation site (indicated by MGCFHS), EF-hand motifs, and C-terminal region (V indicates the last amino acid), which are critical for membrane association. (**E**) Hydrophobicity profile rotated by 75° along the *Y*-axis, providing a different perspective on exposed hydrophobic regions involved in protein-membrane interactions and SOS2 binding.

The structural model of AmSOS3 was generated using Modeller based on the model template of AtSOS3. The predicted monomeric structure of AmSOS3 (Figure 2A) revealed a well-defined arrangement of four EF-hand motifs similar to that of AtSOS3, with Ca^+2^ ions (red) bound to residues within the loop domains of EF-hands. The modeled dimeric structure of AmSOS3 displays a V-shaped conformation similar to AtSOS3. The superimposition of AmSOS3 onto AtSOS3 demonstrates a high degree of similarity, particularly in the dimerization interface (Figure 2B). In the AmSOS3 dimer, the two monomers are stabilized by a hydrogen bond between serine 140 of one monomer and its counterpart in the other monomer (Figure 2C). Sequence analysis showed this serine residue is conserved among 60 orthologs of CBL4 (Figure S1). Furthermore, hydrophobic surface models of AmSOS3 reveal a standard representation of the hydrophobic regions and a 75°-rotated profile (Figure 2D,E), emphasizing the cytoplasmic-exposed domains, including the N-myristoylation site (MGCFHS), the four EF-hands, and the C-terminal region ending with a valine (V) residue. Exposure of these regions is critical for membrane association and interactions with other signaling components, especially SOS2.

In the EF-hand domain, calcium binding depends on six key positions within the loop, labeled X, Y, Z, −X, −Y, and −Z, which together hold the calcium ion in place.

The side chain oxygen donors at positions X, Y, Z, and −Z facilitate Ca^2+^ binding, while the residues at positions −X and −Z contribute to stabilization through internal coordination involving backbone oxygen atoms and water-mediated interactions [16,36].

**Figure 3:**
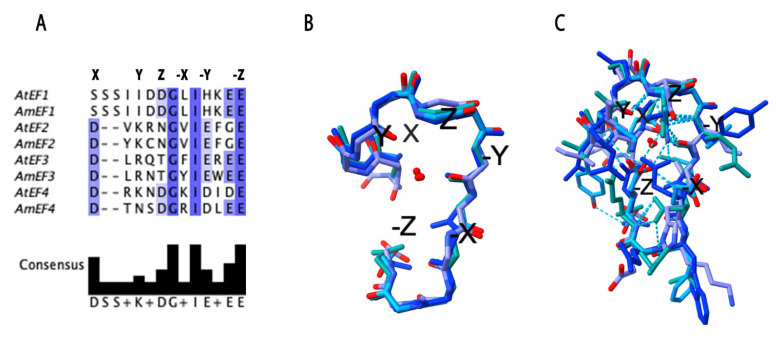
EF-hand domains. (**A**) Sequence alignment of the EF-hand motifs from AmSOS3 and AtSOS3, highlighting the conserved calcium-binding residues. (**B**) Superimposed backbone structures of all four EF-hand motifs of AmSOS3, showing the loop arrangement essential for calcium coordination. (**C**) Atom-level representation of the EF-hand motifs, emphasizing the spatial conservation of key residues and the X, Y, Z/−X, −Y, −Z coordinates, which define structural positioning and functional interactions.

The sequence alignment of the EF-hand motifs from AmSOS3 and AtSOS3 reveals a high degree of conservation with the consensus residues for XYZ and -XYZ highlighted (Figure 3A). These consensus residues are identical for EF-1 and EF-2 to the respective EF-hand domains AtSOS3. However, in EF3 and EF4 of AmSOS3, phenylalanine (F) is replaced by tyrosine (Y) at the −X position, and lysine (K) is replaced accordingly. EF4 also exhibits an additional substitution at the Y position, where lysine (K) is replaced by asparagine (N). Whether these amino acid substitutions have functional implications remains to be determined. In addition to the six consensus residues, the isoleucine (I) residue at the −X + 1 position is also conserved, a feature reported to be important for Ca^2+^ binding between EF-hands [16]. This residue is conserved in 60 CBL4 orthologs (Figure S1). The superimposed models of the backbone structure and an atom-level representation of the four EF-hands show the conserved loop arrangement essential for calcium coordination (Figure 3B,C). Together, these structural insights confirm that AmSOS3 retains the conserved calcium-binding architecture characteristic of SOS3 proteins. While amino acid variations in its EF-hand motifs may reflect adaptive features specific to halophytic environments, their functional relevance remains to be experimentally validated. These findings provide a foundation for future functional studies and biotechnological applications aimed at developing stress-resilient crops.

### 3.3. AmSOS3 Complements sos3-1 Mutantation in Arabidopsis thaliana

**Figure 4:**
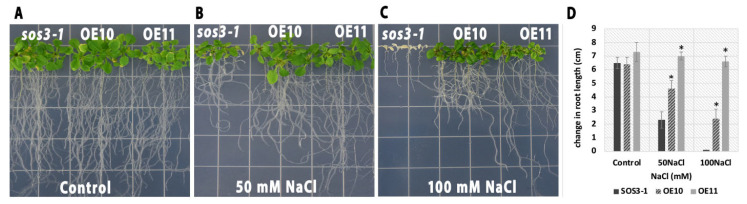
Complementation of the *Atsos3-1* mutant by *AmSOS3*. Growth comparison of the *sos3-1* mutant and th*e AmSOS3*-transformed *sos3-1* plants under normal and salt stress conditions. (**A**) The growth of mutants and transgenic plants in ½ MS agar plates, (**B**) on ½ MS agar plates supplemented with 50 mM NaCl, or (**C**) 100 mM NaCl. (**D**) Root growth analysis of transgenic and mutant plants under both conditions. SD was shown as an error bar. An asterisk indicates statistical significance.

To determine whether AmSOS3 can functionally complement the *sos3-1* mutant phenotype, *sos3-1* mutant *Arabidopsis thaliana* plants were transformed with the pRI 201-AN:35S::AmSOS3 construct, in which *AmSOS3* is under the control of the 35S promoter. The growth of two independent transgenic lines (OE10 and OE11) and non-transformed mutant plants was evaluated under salt stress conditions on ½ MS medium supplemented with either 50 mM or 100 mM NaCl. Under normal conditions, both transgenic and mutant plants exhibited comparable growth (Figure 4A). However, in the presence of 50 mM NaCl, transgenic lines consistently displayed improved growth, while *sos3-1* mutants exhibited severe growth retardation (Figure 4B). At 100 mM NaCl, the *sos3-1* mutants failed to survive, whereas all transgenic plants continued to grow (Figure 4C and Figure S2). The root growth of transgenic plants was greater than that of the *sos3-1* mutants in both conditions (Figure 4D). These results provide compelling evidence that *AmSOS3* is a functional ortholog of *AtSOS3*, fully capable of restoring salt tolerance in the *sos3-1* mutant background, thereby highlighting its critical role in the SOS signaling pathway.

### 3.4. AmSOS3 Enhanced Salt Stress in Arabidopsis thaliana

To assess the endogenous expression patterns of *AmSOS3*, the transcripts levels of three-month-old *Avicennia marina* seedlings were measured. qPCR analysis revealed that endogenous *AmSOS3* expression was higher in the main roots and lateral roots compared to the leaves (Figure S3A). Interestingly, the expression of *AmSOS3* in the lateral roots was induced at 4 and 24 h after 500 mM NaCl treatment, while the expression levels were unchanged in the main roots and leaves (Figure S3B). This pattern may reflect the primary role of roots in sensing and responding to salt stress.

**Figure 5:**
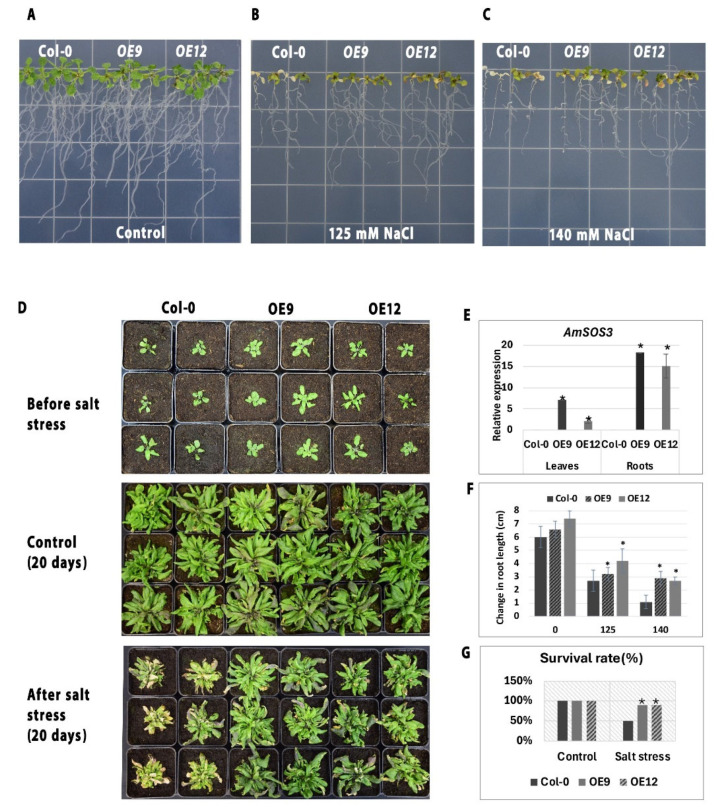
Assessment of AmSOS3 overexpression in *A. thaliana*. (**A**–**C**) Growth response of WT and transgenic lines on plates supplemented with 125 mM or 140 mM NaCl (n = 12). (**D**) Phenotypic comparison of AmSOS3-expressing transgenic plants and wild-type (WT) plants after 20 days of salt stress. (**E**) Quantitative PCR analysis of AmSOS3 expression in roots and leaves of two independent transgenic lines and WT, with three biological replicates. (**F**) Root elongation analysis under salt stress (n = 12). (**G**) Survival rates of transgenic and WT plants following 20 days of salt treatment (n = 12). SD was shown as an error bar. An asterisk indicates statistical significance.

To evaluate whether the heterologous expression of *AmSOS3* confers salt tolerance, wild-type (WT) *Arabidopsis thaliana* plants were transformed with the pRI 201-AN:35S::AmSOS3 plasmid. The expression of *AmSOS3*, under the constitutively active 35S promoter, was confirmed in both roots and leaves of two independent transgenic lines (OE9, OE12) using quantitative PCR. No expression was detected in WT plants (Figure 5E). Although *AmSOS3* was expressed under the constitutive CaMV 35S promoter, *AmSOS3*’s expression was notably higher in roots than in leaves in both lines. Similar patterns of root-preferential expression under the 35S promoter have been documented, as previous studies showed that its activity varies across tissues and developmental stages, with consistently higher expression levels in roots [37,38]. These results confirmed the successful integration of *AmSOS3* in the transformed plants. Importantly, no developmental defects were observed in the transgenic plants. To assess the effect of *AmSOS3* overexpression on salt tolerance, WT, and transgenic lines were subjected to salinity stress by growing them on ½ MS agar plates supplemented with 125 mM or 140 mM NaCl. There were no significant differences in the root length between the transgenic lines and WT plants under non-stress conditions (Figure 5A). In stressed conditions, transgenic lines consistently exhibited better growth and survival than WT plants under both salt concentrations (Figure 5B,C and Figure S4). To further assess the impact of *AmSOS3* overexpression on salt tolerance, transgenic and WT plants were grown in pots and subjected to salinity stress through irrigation with 150 mM NaCl solution. Under control conditions, both transgenic and WT plants exhibited comparable growth (Figure 5D). Upon exposure to salt stress, while WT plants exhibited chlorosis, reduced biomass, and lower survival rates, the transgenic lines maintained better growth and displayed fewer stress symptoms (Figure 5D). Transgenic plants demonstrated improved overall performance under salt stress, with significantly higher survival rates compared to WT (Figure 5G). These results indicate that overexpression of *AmSOS3* enhanced salt stress tolerance in *Arabidopsis*, supporting its functional role in stress adaptation.

### 3.5. AmSOS3 Mitigates Oxidative Stress

**Figure 6:**
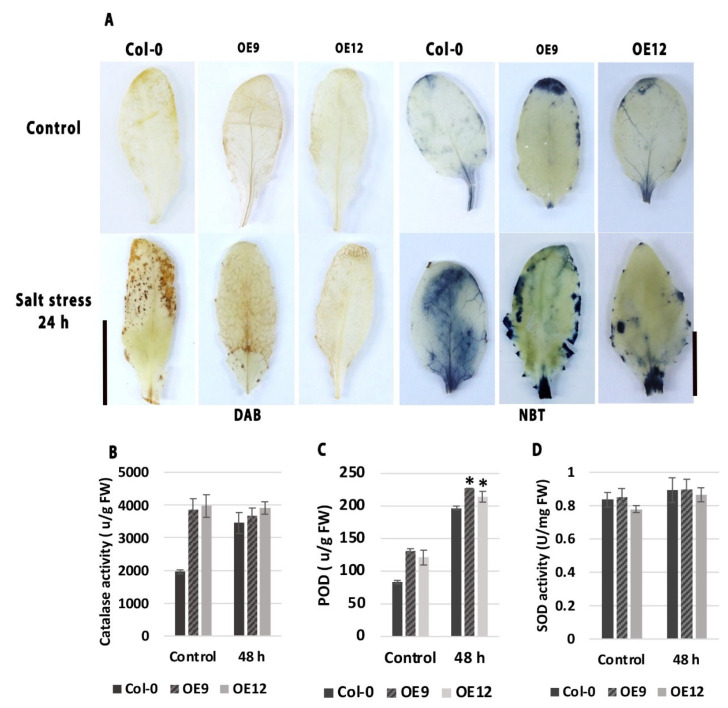
Evaluation of ROS and antioxidant enzyme activity. (**A**) Histochemical staining for superoxide (NBT staining) and hydrogen peroxide (DAB staining) accumulation in WT and *AmSOS3* transgenic lines under control and salt stress conditions. Bars = 1 cm. (**B**) Quantitative antioxidant enzyme activities of catalase (CAT), (**C**) peroxidase (POD), and (**D**) superoxide dismutase (SOD) in WT and transgenic lines exposed to salt stress (n = 3). SD was shown as an error bar. An asterisk indicates statistical significance.

To assess the impact of *SOS3* overexpression on oxidative stress regulation under salt stress, the reactive oxygen species (ROS) accumulation and antioxidant enzyme activities were evaluated. Histochemical staining using nitro-blue tetrazolium (NBT) and 3,3′-diaminobenzidine (DAB) was performed to detect superoxide and hydrogen peroxide (H_2_O_2_) accumulation, respectively. As shown in Figure 6A, WT plants exhibited stronger NBT and DAB staining under salt stress, indicating higher levels of ROS accumulation, a sign of stress response. In contrast, transgenic lines displayed significantly weaker staining, suggesting reduced ROS accumulation and enhanced ROS detoxification. To further investigate the enzymatic mechanisms underlying this response, the activities of catalase (CAT), peroxidase (POD), and superoxide dismutase (SOD) were quantified. These enzymes are key antioxidants essential for neutralizing reactive oxygen species. SOD converts superoxide radicals into H_2_O_2_, which is then detoxified by CAT and POD to protect cells from oxidative damage [39]. CAT activity (Figure 6B) was significantly higher in transgenic lines than in WT under control conditions and remained stable following salt exposure. Similarly, POD activity **(**Figure 6C) was consistently higher in transgenic lines, suggesting an enhanced ROS-scavenging capacity. SOD activity (Figure 6D) showed a moderate increase in response to salt stress across all genotypes, with transgenic lines maintaining more stable levels, reflecting a well-regulated antioxidant defense system. These findings suggest that *AmSOS3* plays a critical role in maintaining cellular homeostasis and regulating oxidative stress responses under salinity stress, thereby improving overall plant performance.

## 4. Discussion

Plants have developed various adaptive mechanisms and signaling pathways to overcome the deleterious effects of abiotic stress. One of the extensively studied mechanisms is the SOS pathway, which plays a crucial role in the response to salt stress by regulating the cellular Na^+^ concentration in root and shoot [15]. The SOS signaling pathway was originally identified in *Arabidopsis thaliana*, and its importance has been established in numerous plants, including crops such as rice, wheat, and maize [40,41,42]. However, the function of the SOS pathway in the halophytic plant, *Avicennia marina*, has not yet been reported. This study explores the molecular structure and function of the SOS3 ortholog from the gray mangrove, *Avicennia marina* (AmSOS3).

Sequence alignment and phylogenetic analysis demonstrated significant similarity, confirming that AmSOS3 is orthologous to AtSOS3/CBL4. AmSOS3 contains four canonical EF-hand domains and an N-myristoylation motif. EF-hand domains are highly conserved calcium-binding motifs found in proteins such as the calcineurin B-like proteins [16]. EF-hands consist of a helix–loop–helix structure, with calcium binding to the loop through several bonds involving six specific conserved residues, which were found to be highly conserved in AmSOS3. Additionally, the isoleucine residue (−X + 1) has been reported to be important for the calcium binding between EF-hands [16], and this residue is conserved in all EF-hands of AmSOS3, further supporting previous findings [16]. This isoleucine residue is conserved across all EF-hand domains of 60 different SOS3 orthologs, confirming its high conservation and functional importance.

The AtSOS3 protein is reported to form a homodimer structure comprising two monomers connected via a hydrogen bond at the 144 position of the serine residue between EF3 and EF4. [16]. The dimeric form is essential for SOS3 function during salt stress [16]. Our predicted model of AmSOS3 supports previous findings, as it also forms a homodimer connected via a serine residue at position 140. The conservation of this serine residue was further analyzed to examine its importance. Sequence analysis of 60 SOS3/CBL4 proteins showed that this serine residue is conserved in all proteins. This further supports the previous findings of dimerization at Ser144 in *Arabidopsis thaliana* and underscores its importance [16]. Notably, serine residues have been reported to be critical for the dimerization of other proteins due to their ability to form strong hydrogen bonds [43,44].

AtSOS3 membrane association requires myristoylation, and S-acylation is essential for nuclear entrance [24]. Although the myristoylation-dependent membrane association is critical for AtSOS3 function, the S-acylation-dependent nuclear localization is not essential for salt tolerance. However, a recent report showed that the nuclear form of AtSOS3 is an important regulator of flowering during salt stress by stabilizing nuclear GIGANTEA (GI) [45]. GI, a regulator of photoperiodic flowering in Arabidopsis, is normally found in both the nucleoplasm and cytoplasm. Under salt stress, cytoplasmic GI is degraded to release SOS2, conferring salt tolerance. Nuclear GI, however, is stabilized by the SOS3 association to facilitate timely flowering [45]. Hence, during salt stress, SOS3 has a dual role, as a vital component of the SOS signaling pathway and a regulator of flowering by stabilization of nuclear GI. The Gly-2 residue for myristoylation and the Cys-3 for S-acylation are conserved in the N-terminal (MGCFHSK) of AmSOS3, suggesting a potential role for AmSOS3 in regulating flowering in gray mangrove. However, the nuclear localization of *AmSOS3* and its potential interaction with GI remain to be tested, and its role in flowering regulation under salt stress, thus, remains hypothetical.

In addition to Arabidopsis, the ability of SOS3 to enhance plant salt tolerance has been reported in several plant species, such as *maize*, *mustard greens*, and *tomato*, highlighting its functional importance [46,47,48]. As salt-tolerant species, halophytes have gained interest as genetic resources for mechanistic investigation and crop improvement. Recent studies have shown that overexpressing SOS3 from the halophytic plants Pongamia and *Tamarix hispida* conferred salt tolerance in *A. thaliana* [21]. In *Avicennia marina*, endogenous *AmSOS3* showed a clear tissue-specific expression pattern, with higher transcript levels in the roots compared to leaves. The induction of AmSOS3 by higher salt concentration in the lateral roots suggest that *AmSOS3* may function predominantly in root tissues, especially lateral roots, where ion uptake and regulation are critical for salt tolerance in mangrove environments.

We cloned and characterized *SOS3* from the gray mangrove, a halophyte inhabiting the UAE coastal regions. *AmSOS3* was transformed into both wild-type and *sos3-1* mutant *Arabidopsis thaliana* plants. Overexpressing *AmSOS3* in *sos3-1* mutant plants reversed their salt hypersensitivity, demonstrating that AmSOS3 functionally complements the *sos3-1* mutation. Furthermore, overexpressing *AmSOS3* in wild-type *Arabidopsis thaliana* conferred salt tolerance. In addition, increased enzymatic activity of ROS-scavenging enzymes such as CAT and POD underscores the elevated detoxification capacity of transgenic plants. These findings suggest that *AmSOS3* plays a critical role in maintaining cellular homeostasis and regulating oxidative stress responses under salinity stress, thereby improving overall plant performance.

## 5. Conclusions

In conclusion, this study is the first to investigate the role of *AmSOS3* in salt stress tolerance. Structural modeling and functional characterization confirmed that *AmSOS3* is an ortholog of *AtSOS3*. Notably, its upregulation significantly enhanced salt tolerance in *Arabidopsis thaliana*. These improvements are likely mediated through calcium-dependent activation of the SOS signaling pathway, stabilization of nuclear GIGANTEA (GI) during stress, and enhanced ROS scavenging. Together, these findings suggest that *AmSOS3* plays a multifaceted role in coordinating stress signaling, ion homeostasis, and may play a role flowering regulation under salinity stress.

## Figures and Tables

**Table 1:** Primer sequences used for amplification and expression analysis of AmSOS3.

**Primers used to amplify the AmSOS3 cDNA**	
**Primer Type**	**Sequence (5′→3′)**
Degenerate primers	F: CSACYRTTCTTGCTGCYGAG
	R: CCTTSAGATATGGRAGRGTCA
RACE primers	F: GATTACGCCAAGCTTTCTTCCACCCCAAGACGGCCACATC
	R: GATTACGCCAAGCTTGAGGTTCGCTCGCTTGCTGTTCCTG
Full-length specific primers	F: CATATGCTCCGATGGGCTGCTTTC
	R: GTCGACATTTACACGTCTGAGTCTTCAACTTC
**Primers used in real-time RT-PCR analysis**	
*SOS3*	F: GGATGTCGTTGAAATGATCGTGG
	R: TTCCTTCCACTCTTCTAGGTCGA
*ACTIN*	F: TGGTGATGAAGCTCAGTCCA
	R: TGAGTAGAACTGGGTGCTCC
EF-1a	F: AACGGTGATGCTGGATTTGT
	R: CCACGCTCTTTATGACACCA

## Data Availability

The cDNA sequence of AmSOS3 was deposited in NCBI-GenBank database under the accession number: PV555410.

## Supplementary Materials

The following supporting information can be downloaded at: https://www.mdpi.com/article/10.3390/cells14120935/s1, Figure S1: Sequence alignment of AmSOS3 against 60 different orthologs; Figure S2: Complementation of the *Atsos3-1* mutant by *AmSOS3*; Figure S3: Expression analysis of *SOS3* in different tissues of *Avicennia marina* under control and salt-stress conditions; Figure S4: Growth response of WT and transgenic lines on plates supplemented with 125 mM or 140 mM NaCl (n = 12).

## Abbreviations

The following abbreviations are used in this manuscript:

SOS	Salt Overly Sensitive (pathway)
CBL	Calcineurin B-like
EF-hand	Helix–Loop–Helix Calcium-Binding Motif
Ca^2^⁺	Calcium Ion
Na⁺	Sodium Ion
H⁺	Proton (Hydrogen ion)
ROS	Reactive Oxygen Species
CAT	Catalase
POD	Peroxidase
SOD	Superoxide Dismutase
DAB	3,3′-Diaminobenzidine
NBT	Nitro-blue Tetrazolium
MS	Murashige and Skoog Medium
CTAB	Cetyltrimethylammonium Bromide
PVP	Polyvinylpyrrolidone
EDTA	Ethylenediaminetetraacetic Acid
PCR	Polymerase Chain Reaction
qPCR/RT-qPCR	Quantitative Real-Time Polymerase Chain Reaction
cDNA	Complementary DNA
ORF	Open Reading Frame
RACE	Rapid Amplification of cDNA Ends
WT	Wild-Type
