# Supplementary material for: SOS3 from Avicennia marina Enhances Salt Stress Tolerance of Arabidopsis thaliana
Source: Cells. 2025 Jun 19;14(12):935. doi: 10.3390/cells14120935 (PMC12191392; doi:10.3390/cells14120935)
Supplement: Supplementary file 1 [file cells-14-00935-s001.zip › cells-3644334-supplementary.pdf]

(Supplementary figures)

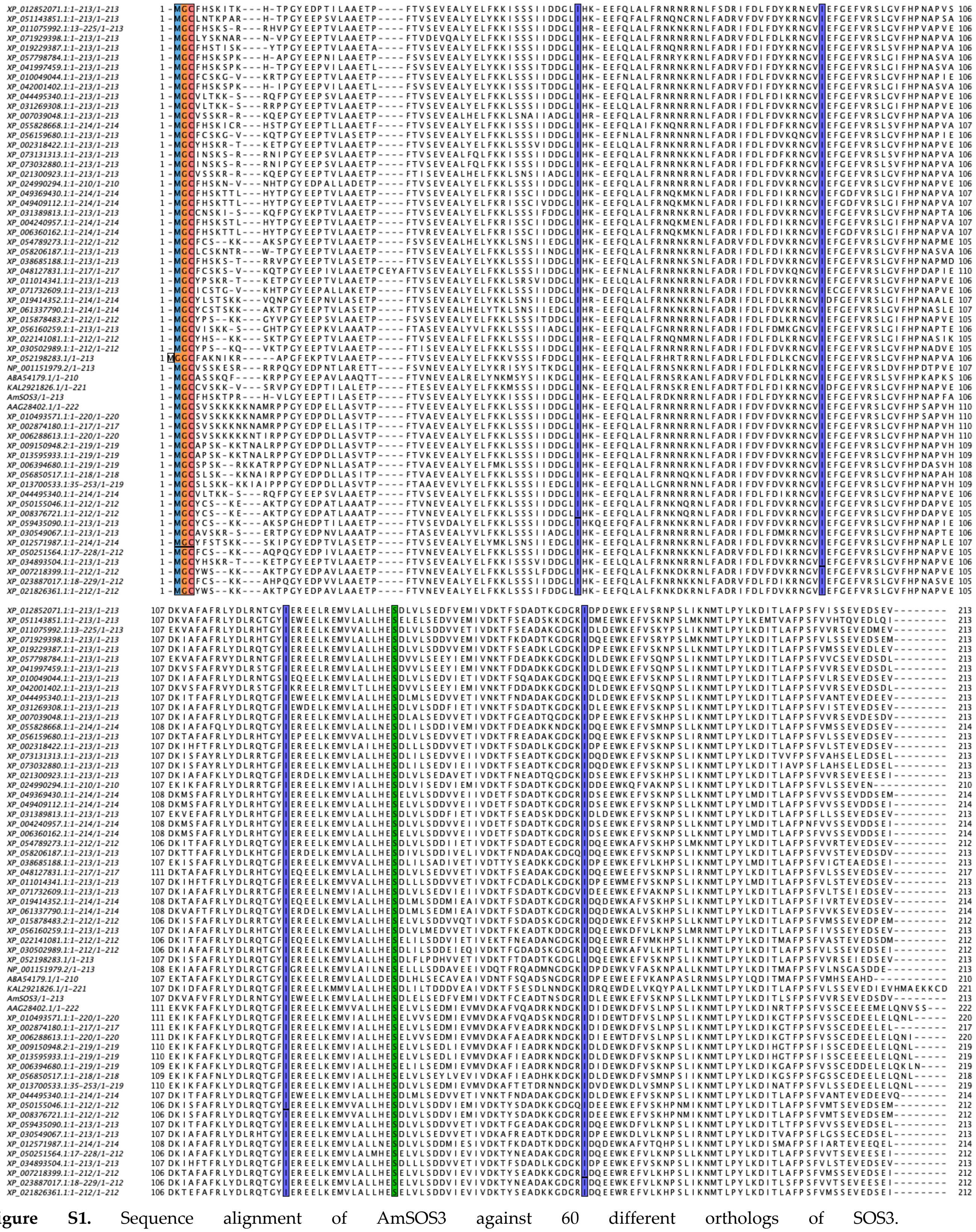

Figure S1. Sequence alignment of AmSOS3 against 60 different orthologs of SOS3.

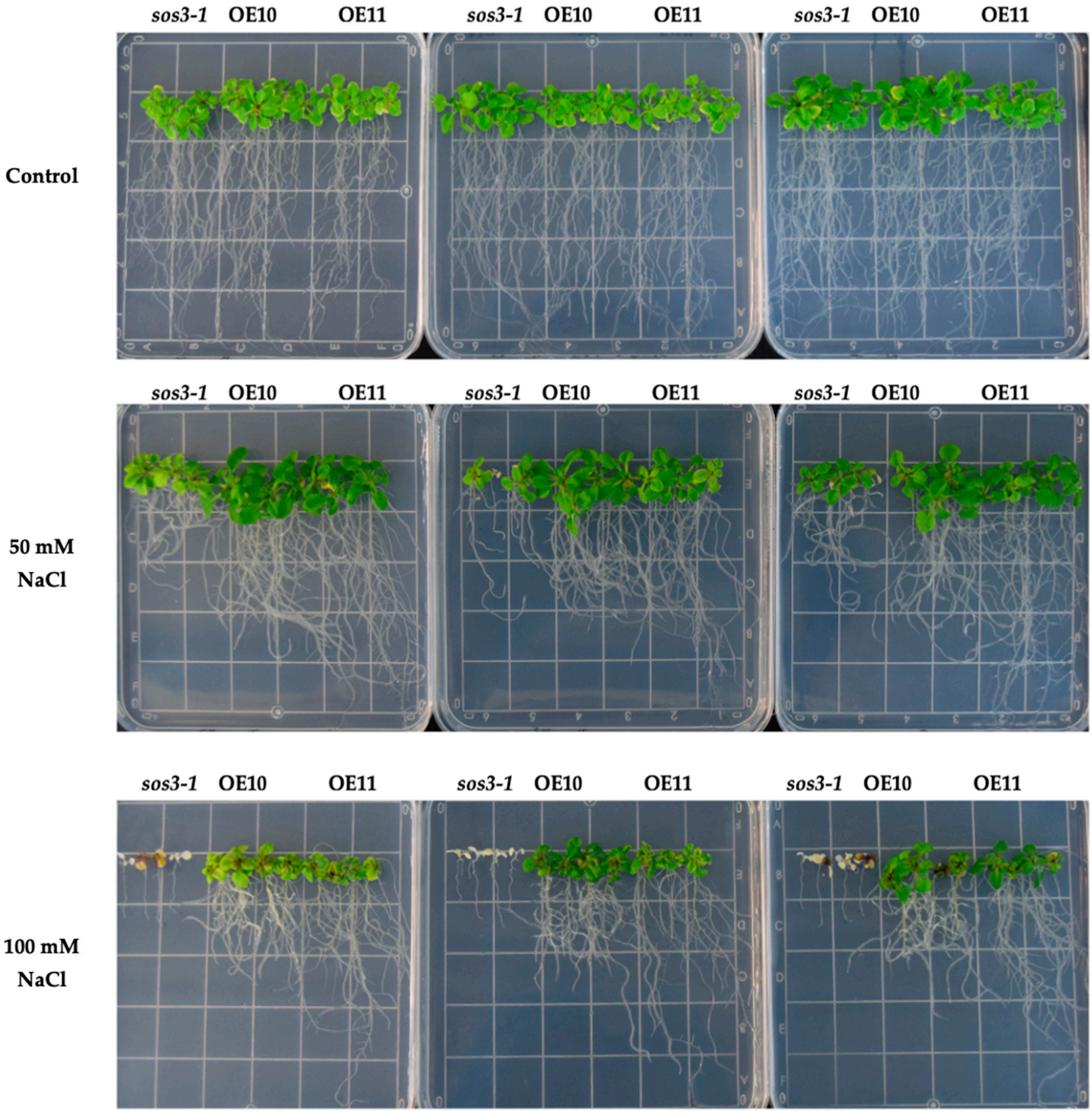

Figure S2. Complementation of the *Atsos3-1* mutant by *AmSOS3*. Growth comparison of the *sos3-1* mutant and the *AmSOS3*-transformed *sos3-1* plants under normal and salt stress conditions (50 or 100 mM NaCl).

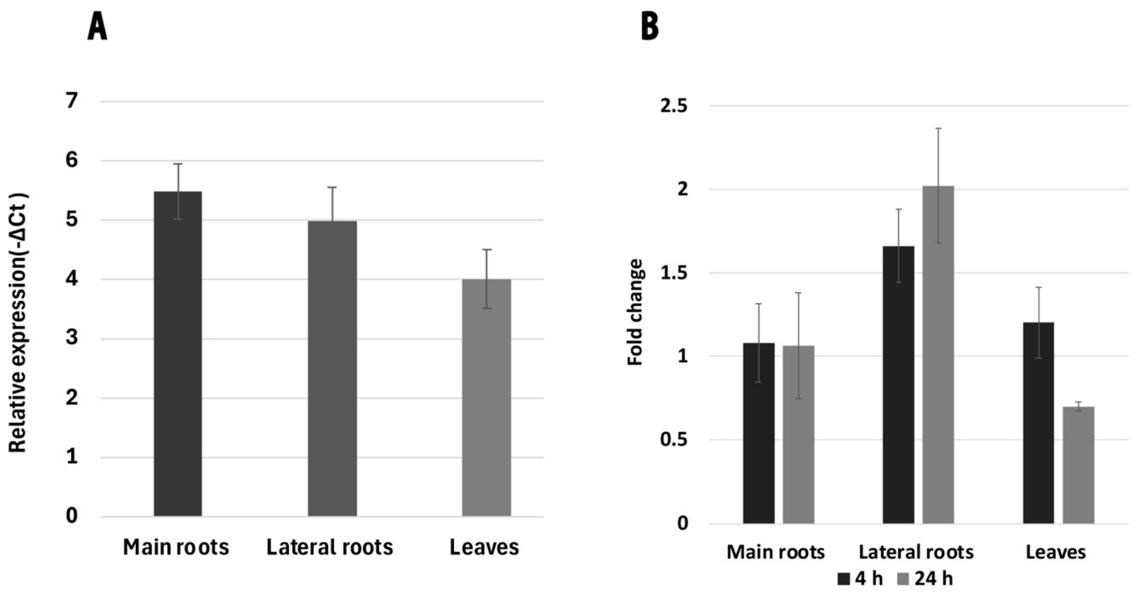

Figure S3. Expression analysis of *SOS3* in different tissues of *Avicennia marina* under control and salt stress conditions. (A) Relative expression of *SOS3* in main roots, lateral roots, and leaves under control conditions. Ct values were obtained using qPCR and normalized to the elongation factor (EF) housekeeping gene. The ΔCt was calculated as Ct_target – Ct_EF for each tissue. (n = 3 biological replicates per tissue).(B) Fold change of *SOS3* expression after 4 h and 24 h salt stress (500 mM NaCl). Expression was normalized to EF and calculated using the ΔΔCt method. (n = 3).

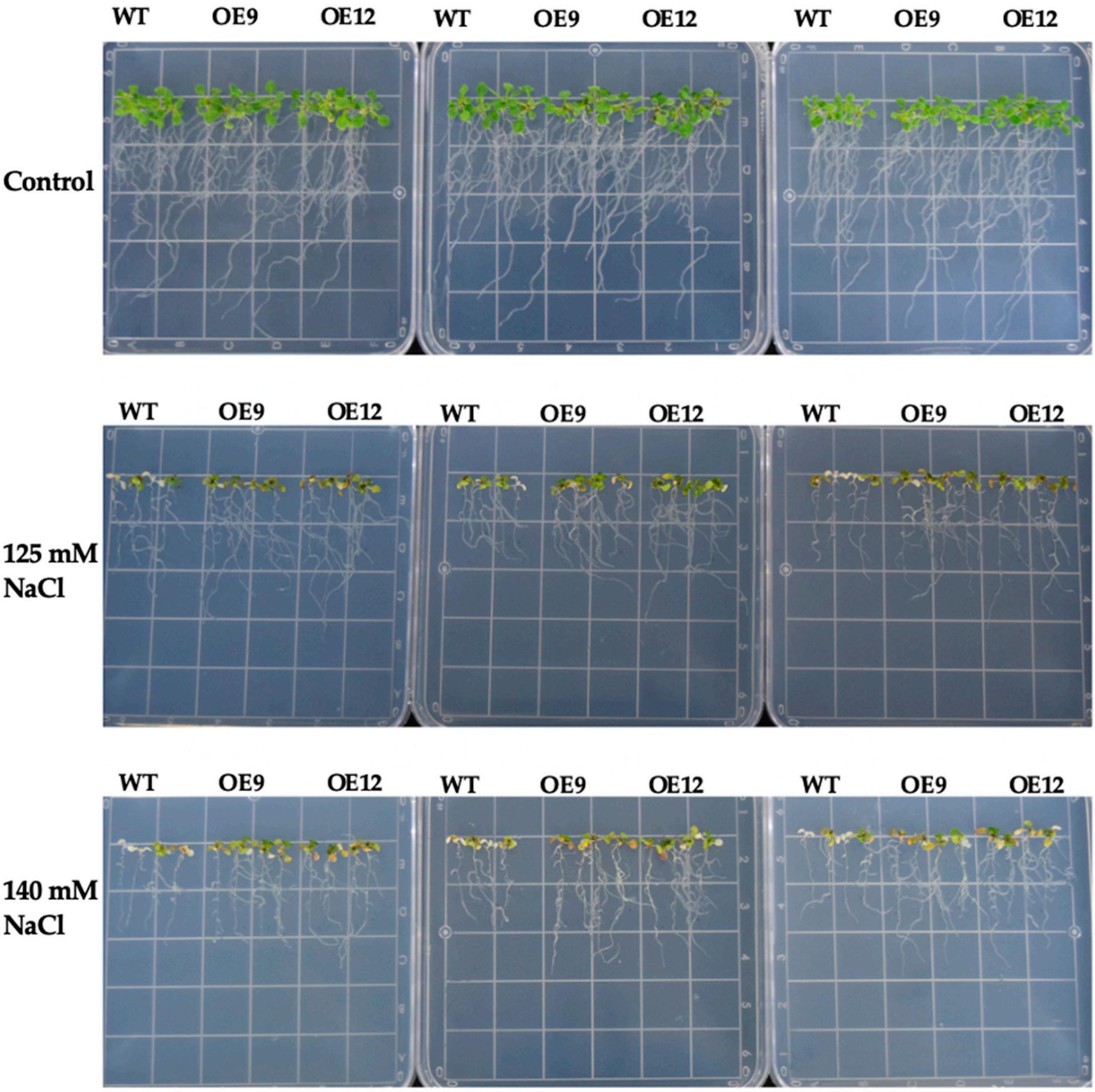

Figure S4. Growth response of WT and transgenic lines on plates supplemented with 125 mM or 140 mM NaCl (n = 12).
